# Draft Genome Sequence of *Lactobacillus crispatus* C25 Isolated from Chicken Cecum

**DOI:** 10.1128/genomeA.01223-16

**Published:** 2016-11-03

**Authors:** Morvarid Rezvani, Mary Mendoza, Matthew D. Koci, Caitlyn Daron, Josh Levy, Hosni M. Hassan

**Affiliations:** a Prestage Department of Poultry Science, North Carolina State University, Raleigh, North Carolina, USA; b Microbiology Graduate Program, North Carolina State University, Raleigh, North Carolina, USA; c RTI International, Research Triangle Park, North Carolina, USA

## Abstract

Lactic acid bacteria are important members of the gut microbiota of humans and animals. Here, we present the genome sequence of *Lactobacillus crispatus* strain C25, originally isolated from the cecum of 4-week-old chicken fed a standard diet. This isolate represents a potential probiotic strain for poultry.

## GENOME ANNOUNCEMENT

Lactobacilli are members of the lactic acid bacteria (LAB); they are found in diverse environments, including plants, fermented foods, oral cavities, and the gastrointestinal tracts of humans and animals. The genus *Lactobacillus* is one of the largest in the LAB group, and the genome sequence of a large number (~174 species) are available (http://www.ncbi.nlm.nih.gov/genomes/lproks.cgi).

*Lactobacillus crispatus* has previously been identified among the vaginal microbiota (1, 2) and the intestine of chickens (3, 4). *L. crispatus* strain C25 was isolated from the cecal microbiota of 4-week-old female commercial white leghorn laying-type chicks (W-36, Hy-line North America, LLC, Mansfield, GA, USA). The birds were housed in climate-controlled HEPA-filtered isolation units (934-1 WP from Federal Designs, Inc., Comer, GA, USA) and were fed *ad libitum* a standard corn-soybean starter diet (NC State Feed Mill). The birds were maintained and euthanized according to a protocol approved by the Institutional Animal Care and Use Committee (OLAW no. A3331-01). The cecal content were collected from bird number 337 and enriched on solid (1.5% agar) modified glucose-free MRS media containing 0.5% purified β (1–4) galacto-oligosaccharide (from Jose Barcena-Bruno) under anaerobic conditions (10% H_2_, 5% CO_2_, and 85% N_2_) using a Coy anaerobic chamber (Coy Lab Products, Grass Lake, MI, USA). The isolate was selected based on its physiological and morphological properties (i.e., Gram-positive stains, non-sporeformer, rod-shaped, production of acid from glucose/lactose, acid coagulation of skim milk, inability to break down hydrogen peroxide, and 16S rRNA gene sequencing). The purified isolate was maintained at −80°C in MRS with 25% glycerol. DNA was extracted from cells, which were grown anaerobically for 20 h in MRS media, using the Promega Wizard Genomic DNA purification kit (Promega Corporation, Madison, WI, USA).

A paired-end library was created for *L. crispatus* C25 with an average insert size of 251 bp. The library was sequenced on an Illumina MiSeq (Illumina, San Diego, CA, USA) at Argonne National Laboratory (Lemont, IL, USA). Modal *k*-mer coverage was 1,060×. After error correction, reads were assembled using MIRA version 4.9.5 (open source: http://genome.cshlp.org/content/14/6/1147.full). The final reported coverage was 79×. After assembly, contigs with less than 20× coverage or length of less than 200 bp were discarded. The length of the draft genome is 2,341,728 bp with a G+C content of 36.8%.

A phylogenetic tree was built using other *Lactobacillus* genomes for comparison (5). The results placed C25 with *L. crispatus*, giving evidence that this isolate was indeed the expected species and that the assembly was of high quality. In addition, we assessed assembly quality by comparing the known metabolism of this strain to both hand- and RAST-annotated functionality (5). The combination of the correct phylogenetic results and correctly predicted metabolism gives sufficient confidence in the subsequent use of these assemblies for biological discovery.

The draft genome was annotated using the NCBI Prokaryotic Genome Annotation Pipeline (http://www.ncbi.nlm.nih.gov/genome/annotation_prok).

### Accession number(s).

The genome sequence of *L. crispatus* C25 was deposited in GenBank under the accession number MCJG00000000.
